# Platform for the rapid construction and evaluation of GPCRs for crystallography in *Saccharomyces cerevisiae*

**DOI:** 10.1186/1475-2859-11-78

**Published:** 2012-06-13

**Authors:** Mitsunori Shiroishi, Hirokazu Tsujimoto, Hisayoshi Makyio, Hidetsugu Asada, Takami Yurugi-Kobayashi, Tatsuro Shimamura, Takeshi Murata, Norimichi Nomura, Tatsuya Haga, So Iwata, Takuya Kobayashi

**Affiliations:** 1 Iwata Human Receptor Crystallography project, ERATO, JST, Yoshidakonoe-cho, Sakyo-ku, Kyoto, 606-8501, Japan; 2 Graduate School of Pharmaceutical Sciences, Kyushu University, 3-1-1 Maidashi, Higashi-ku, Fukuoka, 812-8582, Japan; 3 Department of Medical Chemistry and Cell Biology, Kyoto University Faculty of Medicine, Yoshidakonoe-cho, Sakyo-ku, Kyoto, 606-8501, Japan; 4 Institute for Biomolecular Science, Faculty of Science, Gakushuin University, 1-5-1 Mejiro, Toshima-ku, Tokyo, 171-8588, Japan; 5 Membrane Protein Crystallography Group, Division of Molecular Biosciences, Imperial College London, London, SW7 2AZ, UK; 6 Japan Science and Technology Agency, Core Research for Evolution Science and Technology (CREST), Kyoto University Faculty of Medicine, Kyoto, 606-8501, Japan

**Keywords:** G-protein coupled receptor, Membrane protein, High expression, Screening, Receptor variants, Structural study, *Saccharomyces cerevisiae*

## Abstract

**Background:**

Recent successes in the determination of G-protein coupled receptor (GPCR) structures have relied on the ability of receptor variants to overcome difficulties in expression and purification. Therefore, the quick screening of functionally expressed stable receptor variants is vital.

**Results:**

We developed a platform using *Saccharomyces cerevisiae* for the rapid construction and evaluation of functional GPCR variants for structural studies. This platform enables us to perform a screening cycle from construction to evaluation of variants within 6–7 days. We firstly confirmed the functional expression of 25 full-length class A GPCRs in this platform. Then, in order to improve the expression level and stability, we generated and evaluated the variants of the four GPCRs (hADRB2, hCHRM2, hHRH1 and hNTSR1). These stabilized receptor variants improved both functional activity and monodispersity. Finally, the expression level of the stabilized hHRH1 in *Pichia pastoris* was improved up to 65 pmol/mg from negligible expression of the functional full-length receptor in *S. cerevisiae* at first screening. The stabilized hHRH1 was able to be purified for use in crystallization trials.

**Conclusions:**

We demonstrated that the *S. cerevisiae* system should serve as an easy-to-handle and rapid platform for the construction and evaluation of GPCR variants. This platform can be a powerful prescreening method to identify a suitable GPCR variant for crystallography.

## Background

G-proteincoupled receptors (GPCRs), which represent the largest family of integral membrane proteins, play pivotal roles in mediating signal transduction events in response to ligands such as peptides and amines. GPCRs are major therapeutic drug targets and represent ~ 30% of the market share of all prescription drugs [1]. Although the high-resolution 3D structures of the target GPCRs provide good initial models for drug design, difficulties in expression and purification have been a major bottleneck for structural study. Large quantities of high-quality pure protein are generally required for X-ray crystallography. With the exception of rhodopsin [2-4], which is naturally abundant and can be isolated from rod outer membranes in the eyes, GPCRs generally are not sufficiently abundant to be isolated from their endogenous tissues. Therefore, overexpression in a heterologous host is needed. Various types of hosts have been evaluated for use in GPCR expression, including bacteria, yeast, insect, and mammalian cells, as well as cell-free systems [5,6]. However, only a limited number of GPCRs have been successfully expressed and purified on a large scale. One reason for that may be their instability, which is most likely due to their dynamic activity in the membrane. Recent successes in structure determination have demonstrated the importance of stabilizing receptor in order to achieve high expression and/or facilitation of crystallization [7-13].

Because it is almost impossible to predict what modifications will improve the expression and/or stability of receptors, suitable variants must be selected from the pool of possible variants by trial and error. To facilitate structural studies of GPCRs, a screening system is required that will enable rapid selection of variants. Insect cells have been used as a successful host for structural study, but the screening is laborious and time consuming. *E. coli* has recently been used to screen the thermally stable GPCR variants of turkey β1 adrenergic receptor (tADRB1) [14], human adenosine A2a receptor (hADORA2A) [15,16], and rat neurotensin receptor 1 (rNTSR1) [17]. In addition the crystal structures of the stabilized variants were determined for tADRB1 and hADORA2A [18,19]. However, only a limited number of functionally expressed receptors have been successfully generated in *E. coli*[20].

Yeast is a more preferred host for the expression of GPCRs than *E. coli.* Yeast has a protein quality control system similar to that of mammalian cells, which enables numerous posttranslational modifications and correct disulfide formation of mammalian membrane proteins. This similarity may lead to more functional expression of GPCRs in yeast [21]. *S. cerevisiae* in particular is stable for protein expression, easy to manipulate, and quick to proliferate. *S. cerevisiae* has been extensively tailored for the screening of functional GPCR mutants [22]. In addition, many GPCRs can be as highly expressed in yeast as in mammalian cells [21,23].

We previously established a GFP-based pipeline for the expression and purification of non-GPCR membrane proteins in *S. cerevisiae*[24,25]. *S. cerevisiae* permits the rapid cloning of genes of interest into the 2-μ plasmid by homologous recombination, enabling the direct expression and evaluation of the proteins. The amount and integrity of the target membrane protein can be estimated from the whole-cell fluorescence and in-gel fluorescence after SDS-polyacrylamide gel electrophoresis (SDS-PAGE). Monodispersity, which is a good indicator for purification, can be observed by fluorescence-detection size exclusion chromatography (FSEC) [26]. The gene of a target protein can be transformed with the divided PCR fragments in one step [27]. In the present study, we demonstrate that the platform using *S. cerevisiae* is very useful for the rapid construction and evaluation of GPCR variants for structural study. The stabilized GPCRs in *S. cerevisiae* were expressed at higher levels in *P. pastoris* yeast. Finally, the stabilized human histamine H1 receptor was successfully purified for structural biology study.

## Results

### GFP-based platform for the rapid construction and evaluation of GPCR variants in *S. cerevisiae*

**Figure 1:**
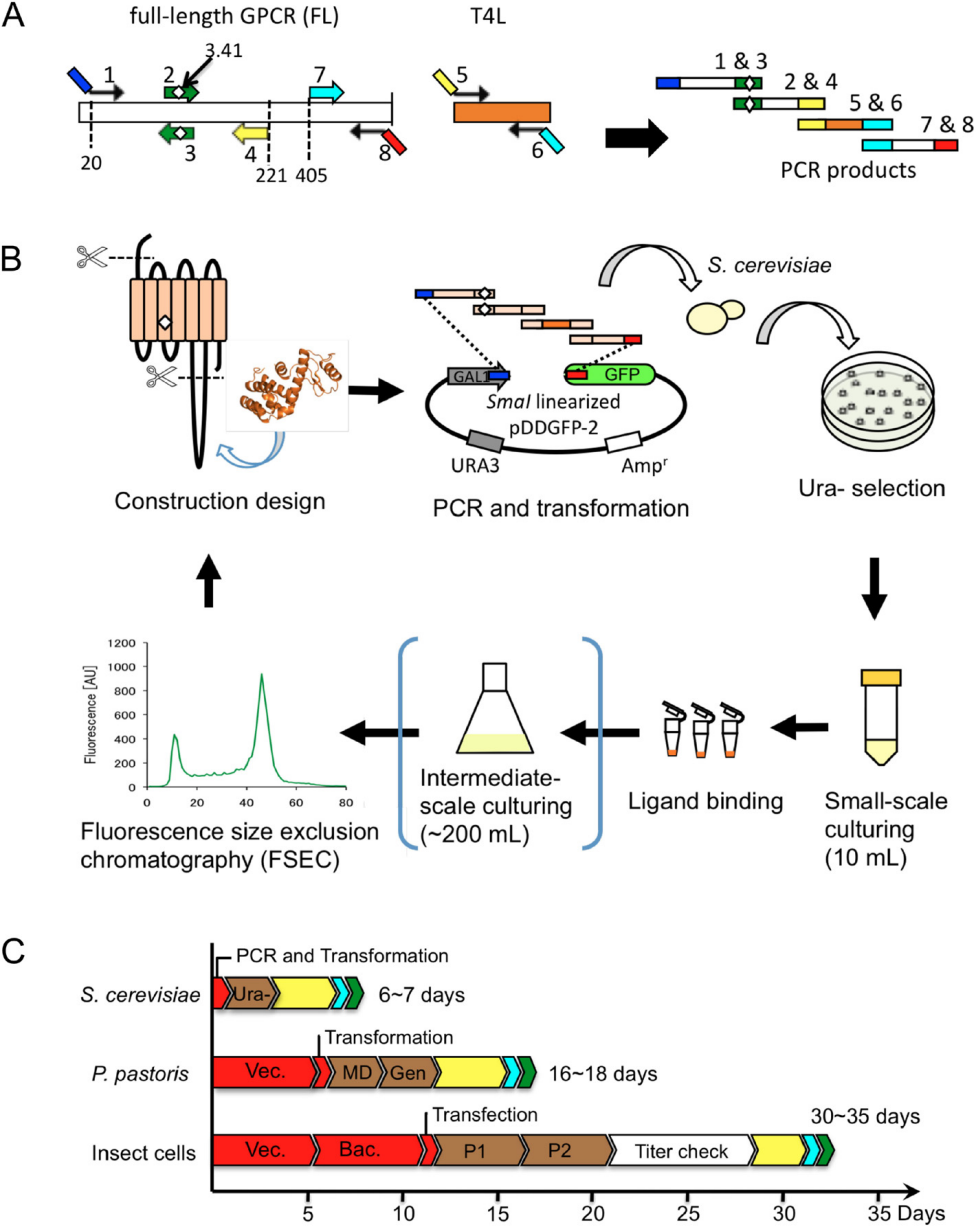
**Overview of the construction and evaluation platform of GPCR variants in *S. cerevisiae. *** (**A**) Primer design of a variant. As an example, the hHRH1 variant with truncation of the N-terminal region, a mutation at the 3.41 position in TM3 and T4L fusion to the i3-loop is shown. The four PCR fragments are generated using the indicated primer pairs from full-length GPCR and T4L ( Additional file 1: Table S1). The same colored overlapping regions were necessary for homologous recombination in *S. cerevisiae*. (**B**) Illustration of a cycle from construction design to evaluation for the GPCR variants. (**C**) Flow and time-scale of the construction and evaluation of GPCR variants in three hosts. Schemes for small-scale culturing are shown in yellow and orange, respectively. Evaluations by ligand-binding assays and FSEC are shown in cyan and green, respectively. Vec.: preparation of expression vector, Bac.: preparation of Bacmid, Ura-: selection on a Ura- plate, MD: selection on a minimum dextrose (MD) plate, Gen: selection on a geneticine plate, P1 and P2: P1 and P2 virus preparation, respectively.

The GFP-based platform using *S. cerevisiae* for the construction and evaluation of GPCR variants is illustrated in Figure 1. The GPCR variants were designed and the genes were generated as PCR fragments (Figure 1A). The 2-μ plasmid named pDDGFP-2, which has a GAL1 promoter, and *S. cerevisiae* strain FGY217 were used [24] (Figure 1B). This plasmid/strain combination resulted in the best expression of membrane proteins. The genes of interest were integrated into the plasmid by homologous recombination in *S. cerevisiae* in one step via introduction of a mixture of linearized plasmid and PCR products. The clone harboring the GPCR variant is selected on an agar plate without uracil (Ura-). After small-scale (10 mL) culturing, the functional expressions are evaluated by radioligand-binding assays. The monodispersity of the detergent-solubilized receptor is assessed by FSEC by detecting the C-terminal GFP, which enables the evaluation without purification. In the present study, the SEC eluate was collected in a 96-well microplate and the fluorescence was detected by a plate reader following the existing protocol [24,25]. Therefore, a relatively large amount of samples (2 ~ 3 mg of total membrane protein) was needed, and the membranes were prepared from an intermediate-scale (200 mL) culture. We have confirmed that a similar result could be obtained from a small-scale (10 mL) culture by using a fluorescence detector at the outlet of the SEC column (data not shown). By omitting the intermediate-scale culturing, this platform enables us to perform a screening cycle within 6–7 days, compared to 16–18 days in *P. pastoris* or 30–35 days in insect cells using baculovirus (Figure 1C).

### Overexpression of full-length GPCRs in *S. cerevisiae*

First, 25 full-length GPCRs were expressed and evaluated using this platform. The integrity of the GPCR-GFP fusions examined by in-gel fluorescence after standard SDS-PAGE [28] indicated that most of the GPCRs were not degraded and appeared as a single major band in the gel ( Additional file 1: Figure S1). Table 1 shows the expression levels of full-length receptors estimated by the GFP fluorescence (total expression) and single-point radioligand-binding assay (functional expression). Without any signal sequence and under standard culture conditions (no additives and at 30 °C), the ligand-binding activities of 12 GPCRs were zero or lower than 0.1 pmol/mg. The N-terminal yeast alpha mating factor signal sequence which improved the ligand-binding activities of GPCRs [29-32]. In the previous report on GPCR expression in *P. pastoris*, supplementation of dimethyl sulfoxide (DMSO) and lowering induction temperature to 20 °C increased the ligand-binding activity of receptor [33]. Then four culture conditions were examined: the presence or absence of 2.5% DMSO and two induction temperatures (20 °C, 30 °C). The functional expression of 24 of 25 GPCRs was increased in *S. cerevisiae* in the optimized conditions. For many receptors, there was little correlation between the intensity of GFP fluorescence and ligand-binding activity, suggesting that the intensity of GFP fluorescence itself is not a good indicator of functional expression in this platform. After these optimizations, 25 GPCRs were functionally expressed. However, the expression level remained insufficient for structural study.

**Table 1:** **Summary of the expression level and functional activity of the 25 GPCRs expressed *S. cerevisiae *** **under different culture conditions in small-scale cultures**

			**α-factor (−)**				**α-factor (+)**			
		**Temp**	**30 °C**		**20 °C**		**30 °C**		**20 °C**	
		**DMSO**	**−**	**+**	**−**	**+**	**−**	**+**	**−**	**+**
Acetylcholine receptor	hCHRM2	TE (mg/L)	1.5	1.5	**1.7**	**1.6**	0.53	0.78	0.62	0.56
		FE (pmol/mg)	0.03	0.12	0.05	0.21	0.08	0.28	**0.30**	**0.66**
Adenosine receptor	hADORA2A	TE (mg/L)	0.20	0.17	0.06	0.06	0.70	0.68	**0.84**	**0.92**
		FE (pmol/mg)	5.3	6.8	2.4	2.4	**24**	**25**	16	20
Adrenergic receptor	hADRB2	TE (mg/L)	**1.5**	**1.6**	0.76	0.93	0.81	1.0	0.91	0.71
		FE (pmol/mg)	0.06	0.07	0.10	0.18	0.36	0.40	**0.52**	**1.1**
Dopamine receptor	hDRD1	TE (mg/L)	0.53	**0.81**	0.75	**0.9**	0.44	0.62	0.54	0.57
		FE (pmol/mg)	**0.07**	0.02	**0.05**	0.01	0.01	0.03	0.01	0.04
	hDRD2	TE (mg/L)	0.21	0.12	0.26	0.24	0.21	**0.32**	**0.34**	0.31
		FE (pmol/mg)	0.37	0.58	0.72	1.57	0.35	1.09	**1.6**	**3.2**
	hDRD4	TE (mg/L)	**2.0**	**2.5**	1.1	1.3	0.78	0.79	0.8	1.3
		FE (pmol/mg)	0.21	0.25	0.19	**0.3**	0.22	**0.34**	0.17	0.17
Histamine receptor	hHRH1	TE (mg/L)	0.95	1.1	**1.6**	**1.7**	0.37	0.53	0.65	0.63
		FE (pmol/mg)	0	0	**0.34**	**0.61**	0	0.15	0	0
	hHRH3	TE (mg/L)	**0.96**	**1.0**	0.61	0.55	0.48	0.64	0.61	0.81
		FE (pmol/mg)	0.4	0.62	0.24	0.11	**0.74**	**0.87**	0.27	0.32
	hHRH4	TE (mg/L)	0.38	0.51	**0.67**	**0.75**	0.33	0.55	0.42	0.52
		FE (pmol/mg)	0.45	0.6	0.16	0.27	**0.68**	**1.1**	0.22	0.15
Neuropeptide Y	hNPY1R	TE (mg/L)	0.11	0.11	0.11	0.11	**0.36**	**0.4**	0.25	0.26
		FE (pmol/mg)	0	0	0	0	0	**0.12**	0.07	**0.21**
	hNPYR2	TE (mg/L)	**0.51**	**0.54**	0.24	0.23	0.18	0.22	0.2	0.19
		FE (pmol/mg)	0	**0.14**	**0.05**	0	0	0	0.03	0
	hNPYR4	TE (mg/L)	1.0	**1.5**	0.91	**1.2**	0.27	0.61	0.24	0.27
		FE (pmol/mg)	0.13	0.18	0.1	0.07	**0.21**	**0.35**	0	0
	hNPYR5	TE (mg/L)	0.31	0.46	**0.82**	**0.89**	0.34	0.51	0.52	0.61
		FE (pmol/mg)	0.04	0.02	**0.07**	**0.13**	0	0.01	0	0
Neurotensin receptor	hNTSR1	TE (mg/L)	0.59	**1.0**	0.87	0.93	0.52	0.8	0.83	**1.1**
		FE (pmol/mg)	0.15	0.15	0.15	0.15	0.32	**0.43**	0.3	**0.78**
	hNTSR2	TE (mg/L)	0.73	1.3	**1.4**	**1.5**	0.65	1.0	0.8	0.89
		FE (pmol/mg)	0.18	**0.21**	**0.26**	0.18	0.19	0.19	0.11	0.16
Opioid receptor	hOPRK1	TE (mg/L)	**0.54**	**0.76**	0.4	0.34	0.24	0.32	0.27	0.29
		FE (pmol/mg)	0	0.08	0.09	**0.16**	0	0.11	0.1	**0.15**
Prostanoid receptor	hPTGER2	TE (mg/L)	0.24	0.16	0.2	0.18	0.28	0.43	**0.49**	**0.48**
		FE (pmol/mg)	0	0	**0.19**	0	0	0.18	0.1	**0.47**
	hPTGER4	TE (mg/L)	0.11	0.15	0.09	0.11	1.4	**1.7**	**1.5**	1.4
		FE (pmol/mg)	0	0	0	0	0	0	**0.4**	0
	hTBXA2R	TE (mg/L)	**0.52**	**0.63**	0.26	0.26	0.21	0.21	0.34	0.32
		FE (pmol/mg)	**0.15**	**0.23**	0.05	0.02	0.03	0	**0.78**	**0.7**
Serotonin receptor	hHTR1B	TE (mg/L)	**0.9**	**1.1**	0.46	0.58	0.34	0.23	0.46	0.46
		FE (pmol/mg)	0.54	0.62	0.71	0.48	0.89	1.1	**2.0**	**1.9**
	hHTR1D	TE (mg/L)	0.34	0.53	**0.67**	**0.76**	0.29	0.3	0.33	0.22
		FE (pmol/mg)	0.06	0.03	0.33	0.46	0.46	0.52	**1.7**	**1.6**
	hHTR5A	TE (mg/L)	0.3	**0.62**	0.48	**0.59**	0.2	0.25	0.23	0.23
		FE (pmol/mg)	0.02	0.18	0	0.07	**0.24**	**0.34**	0	0
Tachykinin receptor	hTACR1	TE (mg/L)	**1.1**	**1.7**	1.1	1.1	0.67	0.83	0.65	0.57
		FE (pmol/mg)	0	0.45	0.14	0.02	**0.60**	**0.59**	0.09	0.15
	hTACR2	TE (mg/L)	1.4	1.7	**2.5**	**2.2**	0.46	0.71	0.96	0.86
		FE (pmol/mg)	0.40	0.85	1.1	**2.9**	0.75	0.65	1.3	**3.8**
	rTACR2	TE (mg/L)	0.11	0.10	0.06	0.04	0.56	0.86	**1.0**	**1.1**
		FE (pmol/mg)	0.25	0.2	0.08	0.06	1.3	1.9	**5.2**	**7.4**

“TE” represents the total expression level estimated from whole-cell GFP fluorescence (mg/L culture). The intensity of 200 μL of 1 μM purified yEGFP in the 96-well plate was 7500 [rfu]. Whole-cell fluorescence was measured in a 200-μL cell suspension by using cultures at the same cell density as the culture. Therefore, the expression level was calculated from: TE [mg/L] = (GFP counts of whole cell [rfu])/(7500*10^6^ [rfu*L/mol])* (molecular weight*10^3^ [mg/mol]). “FE” represents the functional activity from a single-point radioligand binding assay (pmol/mg membrane protein). Conditions highlighted in bold type are the best and second-best conditions for the GFP-based expression level and the functional activity.

### Use of *S. cerevisiae* as a platform for the construction and evaluation of GPCR variants

We generated and evaluated the variants of the three GPCRs shown in Table 2 (hCHRM2, hHRH1, hNTSR1) using the GFP-based platform in *S. cerevisiae*. In addition, the hADRB2 variant (E122W-N187E-Cd), whose structure has been solved as a T4L-fusion [34], was also generated. To construct GPCR variants, the following four variant modules were considered: (1) A truncation of flexible long N- (Nd) or C-terminal residues (Cd), which effectively increases GPCR expression in some reported cases (e.g., ref. [35]). (2) A point mutation at the 3.41 position in transmembrane helix 3 ( Additional file 1: Figure S2A). The numbering is based on the general indexed position in the Ballesteros-Weinstein system [36]. Mutations at this position reportedly increase the thermal stability of hADRB2 [37]. (3) A deletion mutant of a long third intracellular loop (i3-loop) (abbreviated as i3d; Additional file 1: Figure S2B). A long i3-loop potentially becomes a target of degradation or receptor destabilization on the host cell surface. For muscarinic receptors, deletion of the i3-loop results in higher functional expression [38,39]. (4) Replacement of part of the i3-loop by T4 lysozyme (T4L, residues from 2 to 161) ( Additional file 1: Figure S2B); this replacement has been successful in crystallization and structural determination of hADRB2, hADORA2A, hDRD3 and hCXCR4 in cubic phase crystals [8,11-13].

**Table 2:** **GPCR variants constructed and evaluated in *S. cerevisiae *** **in this study**

**GPCR**	**Variant name**	**Deletions and mutations**
hADRB2	FL	full-length ADRB2 (1–413)
	ADRB2mut	ADRB2(1–365), E122W, N187E
hCHRM2	FL	full-length CHRM2 (1–466)
	Nd-i3d	CHRM2 (11–466), i3d(233–380)
	Nd-M112W-i3d	CHRM2 (11–466), M112W, i3d(233–380)
hHRH1	FL	full-length HRH1 (1–487)
	Nd-i3d	HRH1 (20–487), i3d (229–397)
	Nd-T4L	HRH1 (20–487), T4L
	Nd-F116W-i3d	HRH1 (20–487), F116W, i3d (229–397)
	Nd-F116W-T4L	HRH1 (20–487), F116W, T4L
hNTSR1	FL	full-length NTSR1 (1–418)
	Nd-Cd	NTSR1 (43–385)
	Nd-T4L-Cd	NTSR1 (43–385), T4L
	Nd-L157W-Cd	NTSR1 (43–385), L157W
	Nd-L157W-T4L-Cd	NTSR1 (43–385), L157W, T4L

NTSR1 (43–385) means that the N- and C-terminal residues are truncated, the receptor starts from residue 43, and the receptor ends with residue 385. E122W, M112W, F116W and L157W are the mutations to tryptophan at position 3.41. Deletion of the i3-loop from position 229 to 297 is indicated as i3d (229–297). T4L indicates the fusion of the T4 lysozyme sequence between TM5 and TM6.

**Figure 2:**
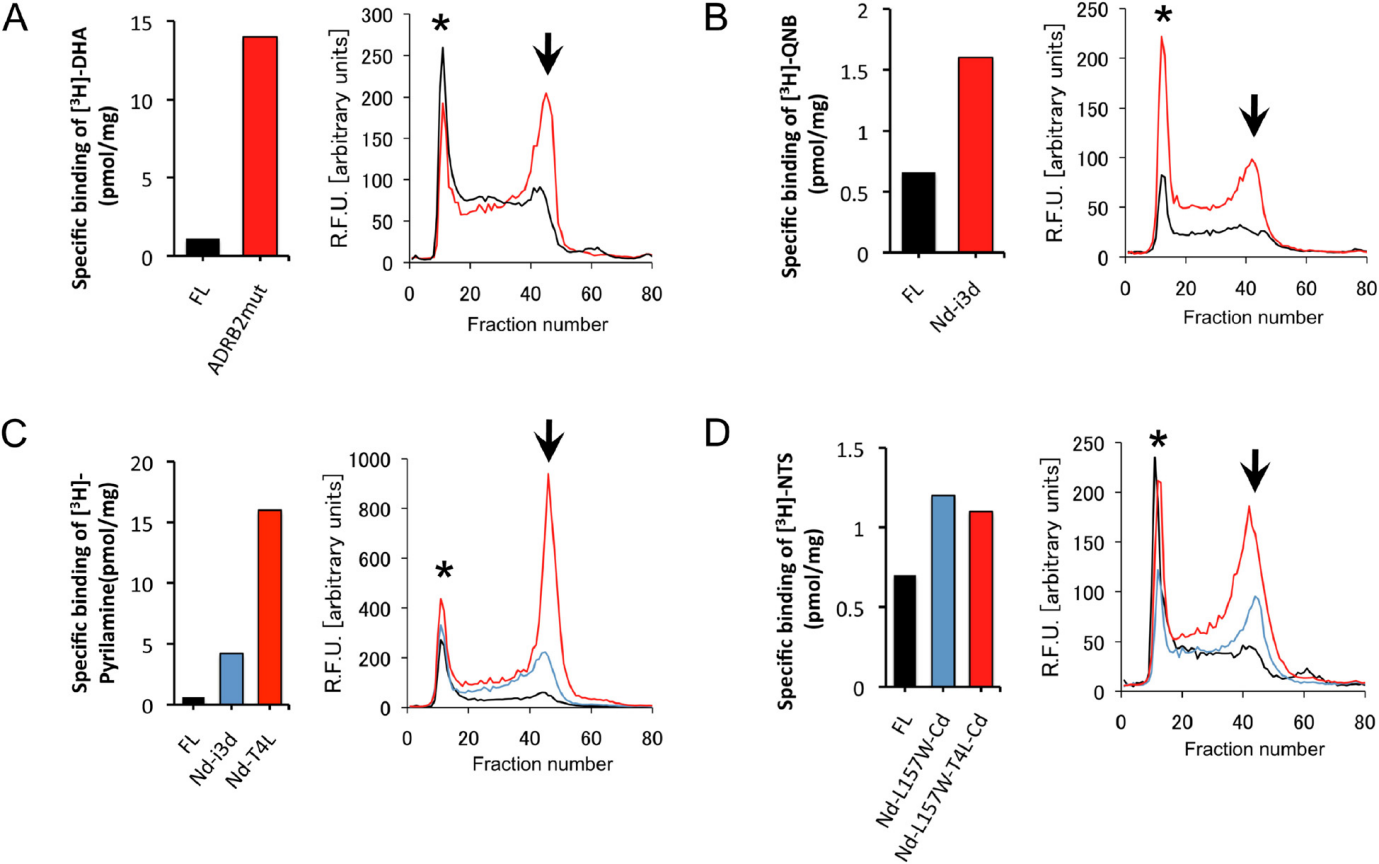
**Specific binding activity (left) and FSEC profile (right) of the full-length and improved variants expressed in *S. cerevisiae. *** (**A**) hADRB2, (**B**) hCHRM2, (**C**) hHRH1, and (**D**) hNTSR1. FSEC was performed with the Superose 6 10/300 column. The colors of the FSEC chromatogram correspond to those of the binding activity. The void peak is denoted by an asterisk. An arrow indicates the target peak of GPCR-GFP fusion.

After small-scale culturing, the total receptor expressions and functional expressions were determined by GFP fluorescence and radioligand-binding assay, respectively ( Additional file 1: Figure S3). These results showed that the intensity of GFP fluorescence itself is not a good indicator of functional expression. The ligand binding activity and monodispersity of the detergent-solubilized receptor of the variants that portrayed improved functional expression are shown in Figure 2. The hADRB2 variant Cd-E122W-N187E showed a ~ 10-fold increase in ligand-binding activity. The hCHRM2 variant Nd-i3d showed a 2.5-fold increase. The hHRH1 variants Nd-i3d and Nd-T4L displayed 7- and 26-fold increased activity, respectively. In hNTSR1, mutagenesis at the 3.41-position (Nd-L157W-Cd and Nd-L157W-T4L-Cd) increased activity by ~ 1.5-fold. Importantly, the variants that showed improved activity also exhibited improved FSEC profiles after solubilization with the mixed micelle of n-Dodecyl-β-D-maltopyranoside (DDM) and cholesteryl hemisuccinate (CHS), which is commonly used for purification of GPCRs. This indicates that there is a correlation between the improvement in both ligand-binding activity and monodispersity in GPCR variants.

### Expression of the GPCR variants in other hosts

Human HRH1-Nd-T4L variant showed a high expression (16 pmol/mg) and good FSEC profile in *S. cerevisiae*. However, the expression of the other GPCR variants remained too low for purification. If the improved variants could be expressed in another host at higher levels, purification would be facilitated. While insect cells are currently known as the most successful host for GPCR expression for structural study, our recent successes in the structural determination of hHRH1 and human adenosine A2a receptor (hADORA2a) were achieved by using *P. pastoris* as a host cell [40,41]*. P. pastoris* is easier to handle compared to insect cells, and can generate milligram quantities of high-quality mammalian GPCR protein as well as insect cells [42-46]. Therefore, we attempted to express selected GPCR variants in *P. pastoris*.

**Figure 3:**
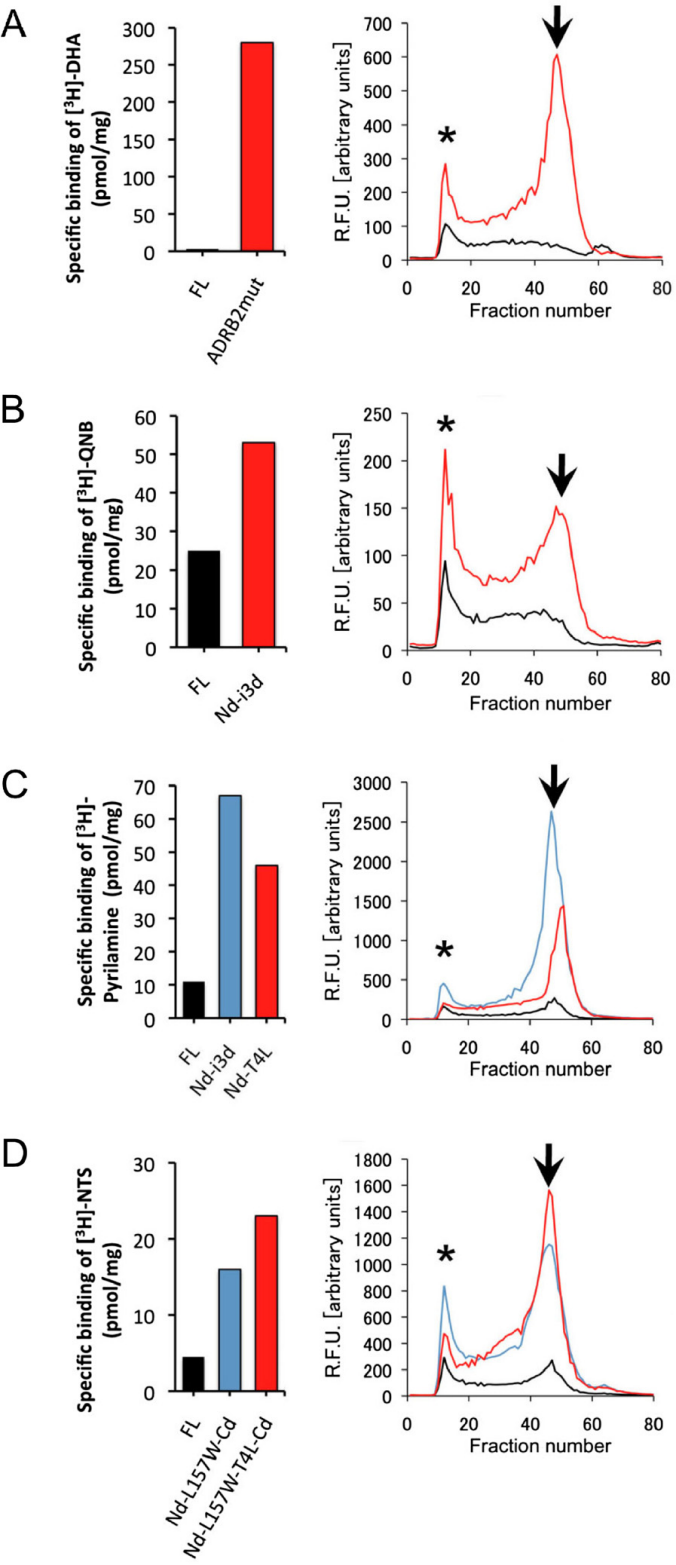
**Evaluation of the GPCR variants expressed in *P. pastoris. *** The specific binding activities (left) and FSEC profiles (right) of full-length GPCRs and the improved GPCR variants expressed in *P. pastoris* are shown. The colors of the chromatogram correspond to those in the binding assays. (**A**) hADRB2, (**B**) hCHRM2, (**C**) hHRH1, (**D**) hNTSR1. FSEC was performed with a Superose 6 10/300 column. The void peak is denoted by an asterisk. The arrow indicates the target peak of GPCR fused to GFP.

Improvements of ligand-binding activity and FSEC profiles of GPCR variants were also observed in *P. pastoris* (Figure 3). The hADRB2 variant E122W-N187E-Cd exhibited a large improvement in ligand-binding activity (80-fold) and a larger peak derived from monodisperse receptor in *P. pastoris*. In the hCHRM2 variant Nd-i3d, improvements in both ligand-binding activity (2-fold) and monodispersity were observed. For hHRH1, substantial improvements in ligand-binding activity and monodispersity in FSEC were observed for both Nd-i3d and Nd-T4L (by 6- and 4-fold, respectively). Although the improvements in ligand-binding activity of the hNTSR1 variants Nd-157W-Cd and Nd-157W-T4L-Cd were only ~ 1.5-fold in *S. cerevisiae*, ligand-binding activity and monodispersity were largely improved in *P. pastoris*. We confirmed that improvements of GPCR variants were also observed in Sf9 insect cells ( Additional file 1: Figure S4). For the receptors in the present study, the expression level in *P. pastoris* was the same or higher than that in insect cells.

### Purification of the GPCR variants

**Figure 4:**
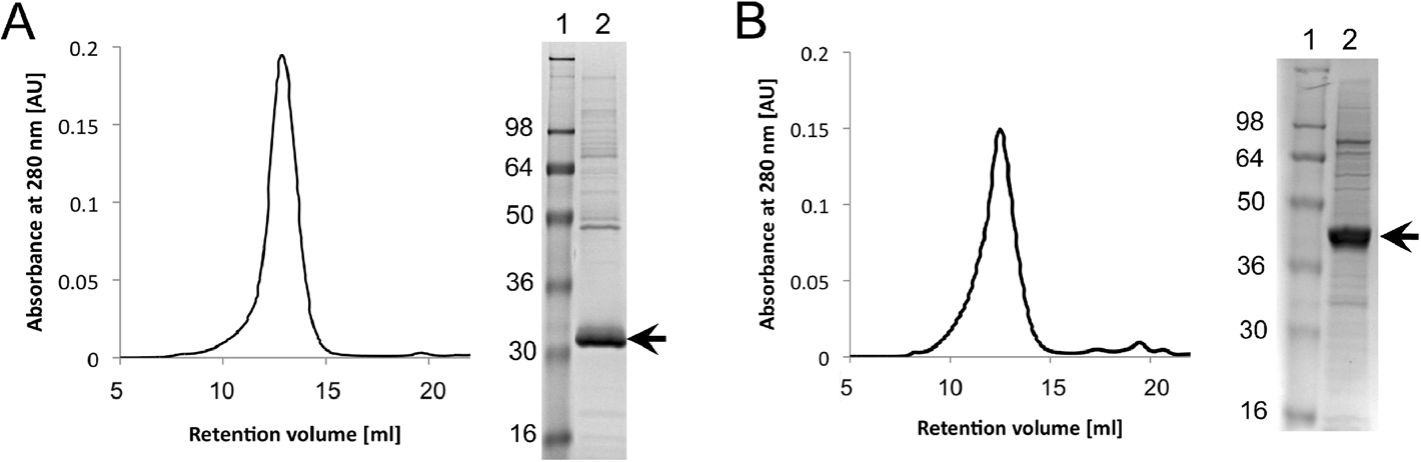
**Purification of the receptor variant.** (**A**) hHRH1-Nd-i3d expressed in *P. pastoris*, and (**B**) hHRH1-Nd-T4L expressed in *S. cerevisiae*. The size-exclusion chromatogram using Superdex 200 10/300 is shown in the left panel, and the Coomassie blue-stained SDS-PAGE gel is shown in the right panel. Lane 1, molecular weight marker. Lane 2, purified receptor variant. Arrows indicate the purified receptors.

The variants, hHRH1-Nd-i3d expressed in *P. pastoris* and hHRH1-Nd-T4L expressed in *S. cerevisiae*, were successfully purified in milligram quantities from a large-scale culture. The yield of functional expression estimated from ligand binding assay were more than 0.3 mg per 1 L culture for hHRH1-Nd-i3d expressed in *P. pastoris* and more than 0.04 mg for hHRH1-Nd-T4L expressed in *S. cerevisiae.* The yield estimated from whole-cell GFP fluorescence intensities were ~5 mg for hHRH1-Nd-i3d expressed in *P. pastoris* and ~1.2 mg for hHRH1-Nd-T4L expressed in *S. cerevisiae.* The final yield after purification of hHRH1-Nd-i3d expressed in *P. pastoris*, hHRH1-Nd-T4L expressed in *S. cerevisiae* were 0.3 ~ 0.4 mg and 0.04 mg per 1 L culture, respectively. These purified GPCR variants showed 90 ~ 95% purity judging from SDS-PAGE and showed a high degree of monodispersity judging from size exclusion chromatography (SEC) with absorbance detection at 280 nm (Figure 4). The SEC profile of purified hHRH1-Nd-T4L expressed in *P. pastoris* was also monodisperse as described in our recent report [47].

## Discussion

Approximately 800 GPCRs have been identified in the human genome. More than 40 receptors are now targeted by drugs, and more than 300 receptors could potentially be future drug targets [48,49]. For a better understanding of ligand recognition and drug design, three-dimensional structures will be needed for each receptor. However, at present, only a handful of GPCR structures have been determined. One major bottleneck that hampers structural study is clearly the expression and purification of functional receptors. As shown in this study and many previous reports on GPCR expression, the expression level and/or stability of wild-type receptors are not sufficient. Therefore, for successful structural study, it is necessary to stabilize the receptors for high-level expression and crystallization. We demonstrated that a platform using *S. cerevisiae* enabled the rapid construction and selection of stabilized GPCR variants.

In this study, we presented examples of receptors that improved ligand-binding activity and monodispersity through the introduction of variant modules. If the initial variant module does not work well, optimizations might be needed to achieve a higher expression, such as changes in the length of the N- or C-terminal truncation and positions of the i3-loop deletion and/or the insertion of T4 lysozyme. Thermal stabilization will be required for the intrinsically unstable receptors, or for receptors that need to be crystallized in a smaller detergent. Thermal stabilizations have been achieved by mutagenesis to tryptophan at position 3.41 in TM3, alanine scanning mutagenesis, or random mutagenesis [14,15,17,37,50]. This platform using *S. cerevisiae* should facilitate such extensive construction screening much more easily and rapidly than in insect cells or mammalian cells. Our results suggest that there is a correlation between the ligand-binding activity and monodispersity; that is, a good FSEC pattern could be a sign of functional expression. Therefore, this platform for engineering stabilized variants could also be applicable to orphan GPCRs, for which functional assays such as ligand-binding assay would be difficult.

Higher expression of the variants than that of the full-length receptors achieved in *S. cerevisiae* was also observed in *P. pastoris* for hADRB2, hCHRM2, hHRH1 and hNTSR1. We confirmed that improvements of GPCR variants were also observed in insect cells. The best host to yield a high quality and quantity of GPCR may depend on the GPCR type. It has been reported that the thermally stabilized NTSR1 variants selected in *E. coli* were also expressed at higher levels in *P. pastoris* and mammalian cells [17]. Therefore, the combination of *E. coli* or *S. cerevisiae* for rapid screening and *P. pastoris* for a large scale of expression could be a dominant strategy for the structural study of GPCR and other membrane proteins.

The purified hHRH1 variants expressed in *S. cerevisiae* and *P. pastoris* showed a high degree of monodispersity analyzed by SEC. The recent structural determinations of hHRH1 and hADORA2a were achieved by using *P. pastoris* expression system [40,41]. These facts strongly suggest the potential of yeast as an expression host, which provide high quality receptor protein enough for crystallography. Nuclear magnetic resonance (NMR) spectroscopy is a promising tool to know the ligand binding to extracellular surface of GPCRs, which is unclear in crystal structure in many cases [51]. Yeast has been used for producing a large amount of isotope-labeled recombinant protein. Our platform can also be a useful system for NMR analysis.

## Conclusions

We demonstrated that the GFP-based *S. cerevisiae* system served as an easy-to-handle and rapid platform for the construction and evaluation of GPCR variants. This platform should be a cost-effective and powerful tool for the extensive screening to identify a highly expressed and stable variant for crystallography. Higher expression of variant achieved in *S. cerevisiae* should be also achieved in other hosts. The combination of *S. cerevisiae* for rapid screening and *P. pastoris* for high expression could be an effective strategy for the structural study of GPCR.

## Methods

### *S. cerevisiae* transformation and small-scale overexpression

The transformation and overexpression in *S. cerevisiae* were basically performed according to the previous method [25]. We here used the 2-μ vector pDDGFP-2 [24] and the vacuolar protease-deficient *S. cerevisiae* FGY217 (*MATα, ura3-52, lys2Δ201,* and *pep4Δ*) [52]. DNA fragments encoding the target GPCRs were amplified with the KOD Plus DNA polymerase (TOYOBO, Tokyo, Japan), with forward and reverse primers containing a 20–30 bp gene-specific region and a 30 bp homologous region [25]. Approximately 30 ng of pDDGFP-2 and 3 μL of 1 ~ 4 PCR fragments of a GPCR (which have a ~30 bp overlapping region with each other) were transformed. Transformants were selected on Ura- plates at 30 °C.

Colonies of transformants harboring the target GPCR were grown in 5 mL of Ura- medium with 2% glucose in 50 mL aerated capped tubes (TPP, Switzerland) at 30 °C overnight. The cultures were diluted to an OD_600_ of 0.12 and cultured in 10 mL of Ura- medium with 0.1% glucose at 30 °C. At an OD_600_ of 0.6, galactose was added to the culture to a final concentration of 2%, DMSO was added as needed, and the temperature was lowered to 20 °C as needed. After shaking for 20–22 h at 30 °C (or 40 h at 20 °C), the cells were harvested, and the cell pellets were resuspended in 700 μL of buffer A (50 mM Tris–HCl, pH 7.5, 5 mM EDTA, 10% glycerol, 0.12 M sorbitol, and complete protease inhibitor cocktail [Roche]). The cell suspensions were diluted 20-fold in buffer A, and whole-cell GFP fluorescence was measured with a SpectraMax M2e microplate reader (Molecular Devices, USA) in a 96-well black plate. Fluorescence at an emission wavelength of 525 nm was measured by using a 515 nm cutoff filter after excitation at 490 nm. Purified yEGFP was used as a standard for estimating overexpression.

Membranes from small-scale (10 mL) cultures were prepared as follows. A yeast cell suspension (700 μL) was transferred to 2 mL tubes containing 500 μL of acid-washed, dry, 425 to 600 μm glass beads (Sigma). Cells were disrupted on a Cutemixer CM-1000 (EYELA, Tokyo, Japan) at 2,500 rpm for 40 min at 4 °C. The samples were examined microscopically to confirm that >90% of the cells were broken. Unbroken cells and debris were pelleted by centrifugation, and the supernatant was transferred into an ultra-centrifuge tube. Yeast membranes were collected by ultracentrifugation at 100,000 g for 30 min at 4 °C. Prepared membranes were snap-frozen in liquid nitrogen and stored at −80 °C, or stored on ice and used within 24 h.

### Intermediate and large-scale overexpression and membrane preparation in *S. cerevisiae*

Yeast clones harboring the target receptor were inoculated in Ura- medium with 2% glucose and grown at 30 °C overnight. The yeast culture was diluted to an OD_600_ of 0.12 in a total volume of 200 mL or more than 1 L of Ura- medium with 0.1% glucose in a 500 mL baffle flask or 2.5 L Tunair flask (Sigma-Aldrich, USA), respectively. The culture was shaken at 250 rpm at 30 °C, and expression was induced by adding galactose to a final concentration of 2% when the culture reached an OD_600_ of 0.6. After induction, the flasks were shaken for 20 h (30 °C cultures) or 40 h (20 °C cultures), and the cells were harvested. Cells were washed once with lysis buffer (50 mM Tris–HCl, pH 7.5, 100 mM NaCl, 5% glycerol, complete protease inhibitor cocktail, and 2 mM EDTA), snap-frozen in liquid nitrogen, and stored at −80 °C.

The intermediate-scale cells were resuspended in the lysis buffer and disrupted in a 50 mL tube using an equal volume of glass beads on a Cutemixer at 2,500 rpm for 40 min at 4 °C. The large-scale cells were disrupted in a 2 L baffled flask using an equal volume of glass beads on an Innova 44R shaker (New Brunswick Scientific Inc., USA) for 60 min at 4 °C. Unbroken cells and debris were removed by centrifugation, the supernatant was transferred to ultracentrifuge tubes, and the membranes were pelleted by centrifugation at 100,000 g for 60 min at 4 °C. The pellet was suspended in buffer containing 50 mM Tris–HCl, pH 8.0, 120 mM NaCl, 20% glycerol and protease inhibitor cocktail, and stored at −80 °C.

### Radioligand-binding assays

Membranes containing each GPCR-GFP were uniformly resuspended by sonication in individual assay buffers ( Additional file 1: Table S2). Membrane proteins were quantified with the bicinchoninic acid (BCA) assay method (Pierce, USA). Membrane suspensions (5–20 μg) were incubated in triplicate with specific radioactively-labeled ligands ( Additional file 1: Table S2) for 1 h at 25 °C. Nonspecific binding was determined in the presence of excess unlabeled ligand. Membranes were trapped on Whatman GF/B filters that were presoaked in 0.3% polyethylenimine. The bound and free radioligands were separated by washing three times with water. The retained radioactivity was measured on an LCS-5100 liquid scintillation counter (ALOKA).

### Fluorescence size exclusion chromatography (FSEC)

Membrane suspensions of GPCRs were solubilized in buffer containing 50 mM Tris–HCl, pH 7.5, 200 mM NaCl, protease inhibitor, and 1% detergent at a final concentration of 3 mg/mL total protein at 4 °C for 1 h with mild agitation. Insoluble material was pelleted by ultracentrifugation at 100,000 g at 4 °C for 30 min. FSEC was performed with a Superose 6 10/300 column (GE Healthcare) on a Biologic Chromatography System (BioRad) with 500 μL of solubilized sample. The column was preequilibrated with running buffer (20 mM Tris–HCl, pH 7.5, 150 mM NaCl, 0.05% DDM/0.01% CHS). Fractions (0.2 mL) were collected in a 96-well microplate from the first 6 mL eluted after sample injection. Fluorescence emission at 525 nm was measured using a SpectraMax M2e plate reader with a 515 nm cutoff filter after excitation at 490 nm.

### Overexpression in *P. pastoris*

Using the GPCR-integrated pDDGFP-2 plasmids as templates, coding regions of the GPCR-GFP fusion proteins were PCR-amplified with a forward primer containing a *BamHI* site (5′-CTA GAA CTA GTG GAT CCA CCA TG-3′) and a reverse primer containing an *EcoRI* site (5′-GCT TGA TAT CGA ATT CCT GCA GTT AAT G-3′). The PCR products were digested with *BamH*I and *EcoR*I and subcloned into the pPIC9K vector. The vector was linearized using *PmeI*. Transformation, clone selection, and small-scale culturing were performed as previously described [53]. The selected transformants were stored in glycerol stocks at −80 °C. Intermediate-scale (200 mL) and large-scale (more than 1 L) culturing were performed under the same conditions as small-scale culturing, with a 500 mL baffled flask and 2.5 L Tuniar Flask (Sigma-Aldrich, USA). *P. pastoris* cells from small- and intermediate-scale cultures were disrupted with glass beads and membranes were prepared in the same way as for *S. cerevisiae*.

### Purification of GPCR variants

Purification of hHRH1-Nd-i3d expressed in *P. pastoris* and hHRH1-Nd-T4L expressed in *S. cerevisiae* was performed according to our previous report [41]. In brief, the membrane containing GPCR-GFP was solubilized by 1% (w/v) DDM/0.2% (w/v) CHS, and the unsolubilized material was separated by ultracentrifugation. The GPCR-GFP fusion protein was purified with TALON IMAC resin (Clontech). The purified protein was concentrated, and then the protein was treated overnight with His-tagged TEV protease (expressed and purified in house). TEV protease and the cleaved His-tagged GFP were removed from the sample by passing the sample through TALON resin and collecting the flow through. All the purification steps were performed in the presence of 100 μM pyrilamine.

## Abbreviations

GPCR: G-protein coupled receptor; GFP: Green fluorescent protein; FSEC: Fluorescence size exclusion chromatography; DDM: n-dodecyl-β-D-maltopyranoside; CHS: Cholesteryl hemisuccinate; T4L: T4 lysozyme; TM: Transmembrane.

## Competing interests

The authors declare that they have no competing interests.

## Authors’ contributions

MS, SI and TK designed the original research project. MS performed the screening of GPCRs using *S. cerevisiae*, the expression in *P. pastoris* and insect cells, the purification of the GPCR vatiants, and wrote the manuscript. HT and HM performed the screening of GPCRs using *S. cerevisiae* and ligand binding assay. HA carried out the Sf9 expression. TY-K carried out the *P. pastoris* expression. TS crystallized the HRH1 variant. TM and NN contributed to the data analysis and interpretation. TH constructed the CHRM2 gene. TK reviewed and wrote the manuscript. MS, SI and TK were responsible for overall project management. All authors provide editorial input.

## Supplementary Material

Additional file 1 Supplementary information. **Table S1** Primers used for the construction of hHRH1 variant (Nd-F116W-T4L). **Table S2** Ligands and conditions for the single point radioligand binding assays. **Figure S1** In-gel fluorescence of 25 GPCR-GFP fusions expressed in *S. cerevisiae.* Arrowheads represent the GPCR-GFP fusion bands and the asterisk represents an endogenous fluorescent ‘background’ protein from S. cerevisiae that migrates at approximately 70 kDa. **Figure S2** Construction design of GPCR variants. (A) Sequence alignments of transmembrane 3 (TM3) of the GPCRs in this study with bovine rhodopsin and human ADORA2A. The number above the sequence is the general indexed position based on the Ballesteros–Weinstein system. The 3.41 position for receptor stabilization is highlighted in yellow. (B) Sequence alignments of TM5, i3-loop, and TM6. The position where the T4 lysozyme sequence is fused is shown in red. To truncate the long i3 loop, the residues shown in blue were connected for each receptor. **Figure S3** Fluorescence intensity and activity of GPCR variants screened in *S. cerevisiae.* Whole-cell GFP fluorescence (arbitrary unit, bar graph) and specific activity of the membrane by radioligand binding assays (black square plot) of full-length GPCRs and GPCR variants constructed in *S. cerevisiae.* (A) hADRB2, (B) hCHRM2, (C) hHRH1, and (D) hNTSR1. **Figure S4** Evaluation of the GPCR variants expressed in Sf9 insect cells. The specific binding activities (left) and FSEC profiles (right) of full-length GPCRs and the improved GPCR variants expressed in Sf9 cells are shown. The colors of the chromatogram correspond to those in the binding assays. (A) hADRB2, (B) hCHRM2, (C) hHRH1, (D) hNTSR1. FSEC was performed with a Superose 6 10/300 column. The void peak is denoted by an asterisk. The arrow indicates the target peak of GPCR fused to GFP. Click here for file
