# Supplementary material for: Platform for the rapid construction and evaluation of GPCRs for crystallography in Saccharomyces cerevisiae
Source: Microb Cell Fact. 2012 Jun 13;11:78. doi: 10.1186/1475-2859-11-78 (PMC3495400; doi:10.1186/1475-2859-11-78)

Supplementary informations

Materials

The cDNAs for human muscarinic acetylcholine receptor subtype 2 (hCHRM2); adenosine A_{2A} receptor (hADORA2A); β_2 adrenergic receptor (hADRB2); dopamine receptor D_1 (hDRD1), D_2 (hDRD2), and D_4 (hDRD4); histamine H_1 (hHRH1), H_3 (hHRH3), and H_4 receptor (hHRH4); neuropeptide Y receptor Y_1 (hNPY1R), Y_2 (hNPY2R), Y_4 (hNPY4R), and Y_5 (hNPY5R); neurotensin receptor 1 (hNTSR1) and 2 (hNTSR2); κ opioid receptor type 1 (hOPRK1); prostaglandin E_2 receptor subtype 2 (hPTGER2) and subtype 4 (hPTGER4); thromboxane A_2 receptor (hTBXA2R); 5-hydroxytryptamine receptor 1B (hHTR1B), 1D (hHTR1D), and 5A (hHTR5A); tachykinin receptor 1 (hTACR1) and 2 (hTACR2); rat tachykinin receptor 2 (rTACR2); and the thermally stabilized turkey β_1 adrenergic receptor mutant were synthesized by TAKARA Bio Inc with α-factor signal sequence in their N termini. (Otsu, Japan). The predicted glycosylation sites were replaced with glutamine or aspartic acid. The *P. pastoris* SMD1163 strain, pPIC9K vector, Sf9 insect cells, pFastBac1 vector, and *E. coli* DH10Bac competent cells were purchased from Invitrogen.

The radio isotope (RI)-labeled ligands, [^{3}H]-CGP12177, -SR48968, -SCH23390, -quinuclidinylbenzilate (QNB), -histamine hydrochloride, -prostaglandin E_2, -angiotensin II, -neuropeptide Y, and -substance P, were purchased from GE Healthcare. [^{3}H]-CP55940, -neurotensin, -diprenorphine, -dihydroalprenolol hydrochloride (DHA), -spiperone, -5-carboxamidotryptamine (5-CT), -SQ29548, and -pyrilamine were purchased from PerkinElmer. [^{3}H]-SB222200 and -ZM241385 were purchased from American Radiolabeled Chemicals Inc. Atropine was purchased from Sigma. ZM241385, CGP12177, angiotensin II, CP55940, butaclamol, pyrilamine, histamine, GR231118, peptide YY, CGP71683, neurotensin, naloxone, prostaglandin E_2, 5-CT, substance P, SR48968, SB222200, and SQ29548 were purchased from TOCRIS. Fos-choline 12 (FC12) and n-dodecyl-β-D-maltopyranoside (DDM) were purchased from Anatrace. Cholesterol

hemisuccinate (CHS) was purchased from Sigma.

Overexpression in Sf9 insect cells

Using the GPCR-integrated pDDGFP-2 plasmids as templates, the coding regions of GPCR-GFP fusion proteins were PCR-amplified with the same primer set as for *P. pastoris*. The PCR products were digested with *BamH*I and *EcoR*I and subcloned into the pFastBac1 vector (Invitrogen). DH10Bac competent cells were transformed with the pFastBac1 plasmid containing the target GPCR-GFP, and recombinant bacmid DNA was prepared with a QIAprep Miniprep kit (QIAGEN). P1 recombinant baculovirus stock was produced by transfecting Sf9 insect cells with the bacmid by using the Bac-to-Bac baculovirus expression system according to the manufacturer's instructions.

Sf9 cells were grown and maintained in a 7:3 mixture of the IPL41 (SAFC Biosciences, KS) and SF-900 II (Invitrogen) media supplemented with 5% fetal calf serum (Invitrogen) and pluronic P-68 (Invitrogen). Penicillin and streptomycin (WAKO, Japan) were added to the medium at final concentrations of 10 mg/L and 10,000 units/L, respectively. The P2 virus stock was prepared by the infection of 1×10^7 cells with P1 virus stock in a 75 cm^2 flask and incubated at 27°C for 5–6 d. After the recombinant baculovirus titer was determined, 1×10^8 cells were infected at a multiplicity of infection (MOI) of 3–5 and cultivated in two 225 cm^2 flasks at 27°C for 72 h to overexpress the GPCR-GFP fusions. Cells were disrupted on ice in PBS buffer containing a protease inhibitor cocktail by using a sonicator (UT-50; SMT Co., Ltd., Japan) with the minimum power output sufficient for cell disruption. The membranes were pelleted at 100,000 *g* for 30 min at 4ºC. The pellet was resuspended by brief sonication in PBS buffer containing a protease inhibitor cocktail, and stored at -80ºC.

Table S1

Primers used for the construction of hHRH1 variant (Nd-F116W-T4L).

No.	
1	5’ TCGACGGATTCTAGAACTAGTGGATCCCCCATGACTACTATGGCTTCTCCACAATTGATGC 3’
2	5’ GCTTCTACTGCTTCTATTTGGTCTGTTTTTATTTTGTG 3’
3	5’ CACAAAATAAAAACAGACCAAATAGAAGCAGTAGAAGC 3’
4	5’ ACAATGTTGTCTAACAGCCTTGTAAATCTTAG 3’
5	5’ CTAAGATTTACAAGGCTGTTAGACAACATTGTAATATATTTGAAATGTTACGTATAGATGAAGGC 3’
6	5’ GCAGCCTTTCTTTCTCTGTTCATATGCAAATACGCGTCCCAAGTGCCAGTTCTAAAC 3’
7	5’ TTGCATATGAACAGAGAAAGAAAGGCTGC 3’
8	5’ AAATTGACCTTGAAAATATAAATTTTCCCCAGATCTAATATGCAAAATTCTCTTAAAAGTCTTC 3’

Table S2. Ligands and conditions for the single point radioligand binding assays.

Receptor	Radioligand	Type of ligand	Conc. [nM]	Cold ligand*	Assay buffer
hCHRM2	^{3}H-QNB	Antagonist	5	QNB	20 mM potassium phosphate pH 7.0
hADORA2A	^{3}H-ZM241385	Antagonist	20	ZM241385	20 mM HEPES pH 7.0, 100 mM NaCl
tADRB1	^{3}H-CGP12177	Antagonist	10	CGP12177	20 mM HEPES pH 7.4, 100 mM NaCl, 12 mM $MgCl_2$
hADRB2	^{3}H-DHA	Antagonist	50	DHA	20 mM HEPES pH 7.4, 100 mM NaCl, 12 mM $MgCl_2$
hDRD1	^{3}H-SCH23390	Antagonist	5	Butaclamol	50 mM Tris-HCl pH 7.4, 5 mM EDTA, 1.5 mM $CaCl_2$, 5 mM $MgCl_2$, 5 mM KCl, 120 mM NaCl
hDRD2, hDRD4	^{3}H-Spiperone	Antagonist	10	Spiperone	50 mM Tris-HCl pH 7.4, 150 mM NaCl
hHRH1	^{3}H-pyrilamine	Antagonist	20	pyrilamine	50 mM Tris-HCl pH 7.4, 5 mM $MgCl_2$
hHRH3, hHRH4	^{3}H-Histamine	Agonist	100	Histamine	50 mM Tris-HCl pH 7.4, 5 mM $MgCl_2$
hNPYR1	^{3}H-NeuropeptideY	Agonist	1	GR231118	50 mM Tris-HCl pH 7.4, 5 mM KCl, 10 mMNaCl, 5 mM $MgCl_2$, 2.5 mM $CaCl_2$
hNPYR2	^{3}H-NeuropeptideY	Agonist	1	PeptideYY	50 mM Tris-HCl pH 7.4, 5 mM KCl, 10 mMNaCl, 5 mM $MgCl_2$, 2.5 mM $CaCl_2$
hNPYR4	^{3}H-NeuropeptideY	Agonist	10	GR231118	50 mM Tris-HCl pH 7.4, 5 mM KCl, 10 mMNaCl, 5 mM $MgCl_2$, 2.5 mM $CaCl_2$
hNPYR5	^{3}H-NeuropeptideY	Agonist	1	CGP71683	50 mM Tris-HCl pH 7.4, 5 mM KCl, 10 mMNaCl, 5 mM $MgCl_2$, 2.5 mM $CaCl_2$
hNSTR1, hNSTR2	^{3}H-Neurotensin	Agonist	10	Neurotensin	50 mM Tris-HCl pH 7.4, 100 mM NaCl
hOPRK	^{3}H-Diprenorphin	Antagonist	10	Naloxone	50 mM Tris-HCl pH 7.4, 10 mM KCl, 10 mM NaCl, 1 mM $MgCl_2$, 2.5 mM $(NH_4)_2SO_4$
hPTGER2	^{3}H-Prostaglandin E_2	Agonist	10	Prostaglandin E_2	20 mM MES pH 6.0, 10 mM $MgCl_2$, 1 mM EDTA
hPTGER4	^{3}H-Prostaglandin E_2	Agonist	5	Prostaglandin E_2	20 mM MES pH 6.0, 10 mM $MgCl_2$, 1 mM EDTA
hTBXA2R	^{3}H-SQ29548	Antagonist	30	SQ29548	20 mM MES pH 6.0, 100 mM $MgCl_2$, 1 mM EDTA
hHTR1B	^{3}H-5-Carboxamide tryptamine (5-CT)	Agonist	100	5-Carboxamide tryptamine	50 mM Tris-HCl pH 7.4, 100 mM NaCl
hHTR1D, hHTR5A	^{3}H-5-Carboxamide tryptamine (5-CT)	Agonist	25	5-Carboxamide tryptamine	50 mM Tris-HCl pH 7.4, 100 mM NaCl
hTACR1	^{3}H-Substance P	Agonist	5	Substance P	30 mM HEPES pH 7.4, 100 mM NaCl, 5 mM $MgCl_2$
hTACR2, rTACR2	^{3}H-SR48968	Antagonist	5	SR48968	50 mM Tris-HCl pH 8.0, 150 mM NaCl

*Non-specific binding was determined in the presence of cold ligand at a 1000-time higher concentration than radio-ligand.

Supplementary Figure Legends

Figure S1 In-gel fluorescence of 25 GPCR-GFP fusions expressed in *S. cerevisiae*. Arrowheads represent the GPCR-GFP fusion bands and the asterisk represents an endogenous fluorescent 'background' protein from *S. cerevisiae* that migrates at approximately 70 kDa.

Figure S2 Construction design of GPCR variants. (A) Sequence alignments of transmembrane 3 (TM3) of the GPCRs in this study with bovine rhodopsin and human ADORA2A. The number above the sequence is the general indexed position based on the Ballesteros–Weinstein system. The 3.41 position for receptor stabilization is highlighted in yellow. (B) Sequence alignments of TM5, i3-loop, and TM6. The position where the T4 lysozyme sequence is fused is shown in red. To truncate the long i3 loop, the residues shown in blue were connected for each receptor.

Figure S3 Fluorescence intensity and activity of GPCR variants screened in *S. cerevisiae.* Whole-cell GFP fluorescence (arbitrary unit, bar graph) and specific activity of the membrane by radioligand binding assays (black square plot) of full-length GPCRs and GPCR variants constructed in *S. cerevisiae*. (A) hADRB2, (B) hCHRM2, (C) hHRH1, and (D) hNTSR1.

Figure S4 Evaluation of the GPCR variants expressed in Sf9 insect cells. The specific binding activities (left) and FSEC profiles (right) of full-length GPCRs and the improved GPCR variants expressed in Sf9 cells are shown. The colors of the chromatogram correspond to those in the binding assays. (**A**) hADRB2, (**B**) hCHRM2, (**C**) hHRH1, (**D**) hNTSR1. FSEC was performed with a Superose 6 10/300 column. The void peak is denoted by an asterisk. The arrow indicates the target peak of GPCR fused to GFP.

Figure S1 (Shiroishi et al.)

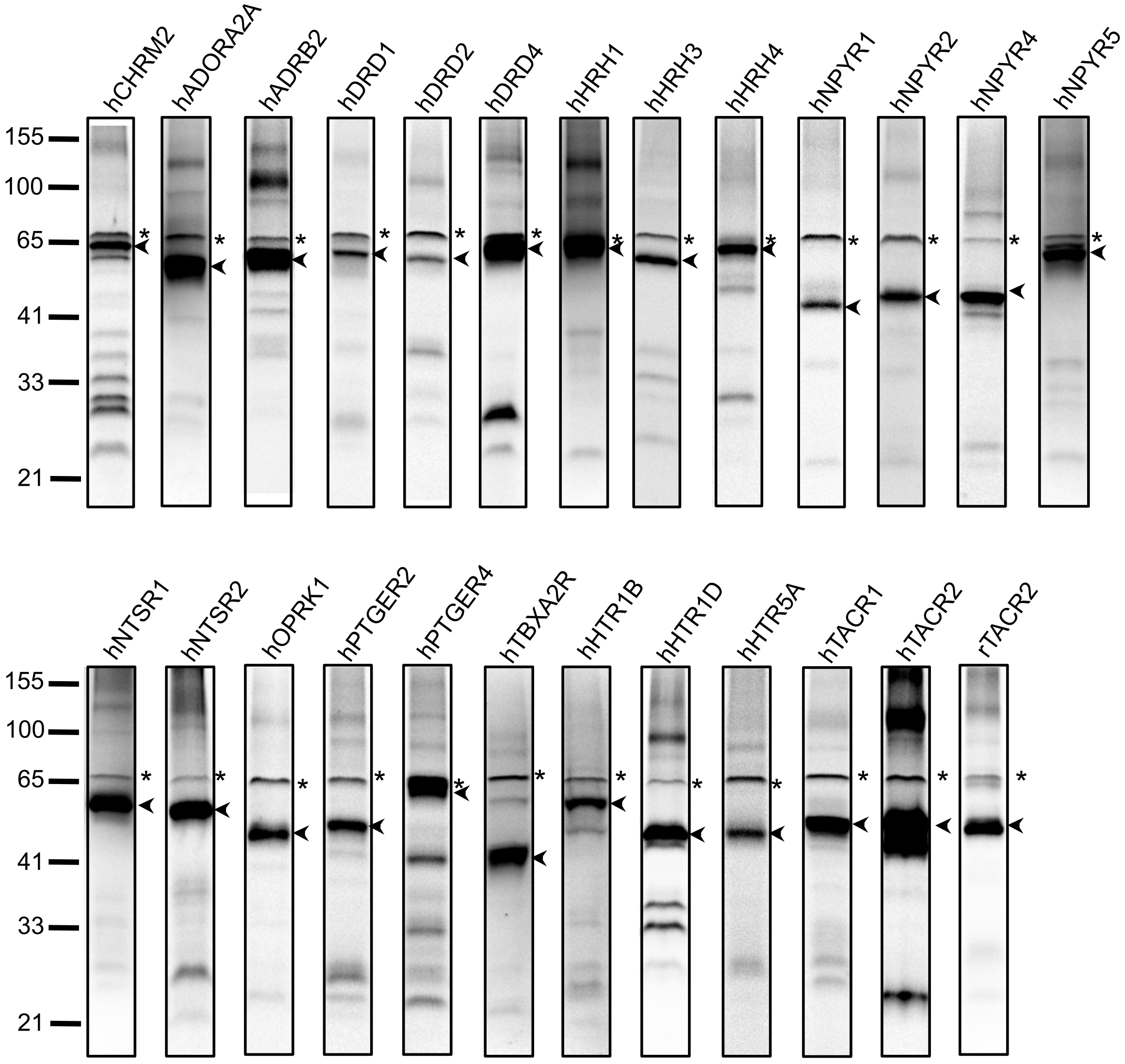

Figure S2 (Shiroishi et al.)

A

```
           3.30      3.40      3.50
             |         |         |
Rho        NLEGFFATLGGEIALWSLVVLAIERY
hADRB2     EFWTSIDVLCVTASIETLCVIAVDRY
hADORA2A   LFIACFVLVLTQSSIFSLLAIAIDRY
hCHRM2     DLWLALDYVVSNASVMNLLIISFDRY
hHRH1      LFWLSMDYVASTASIFSVFILCIDRY
hNTSR1     RGYYFLRDACTYATALNVASLSVERY
           TM3
```

B

```
                   5.50      5.60                                                      6.40      6.50
                     |         |                                                         |         |
Rho        SFVIYMFVVHFIIPLIVIFFCYGQLVFTVKEAAAQQQ----------   ----ESATTQKAEKEVTRMVIIMVIAFLICWLPYAGVA
hADRB2     AYAIASSIVSFYVPLVIMVFVYSRVFQEAKRQLQKIDKSEGRFHVQN   HGLRRSSKFCLKEHKALKTLGIIMGTFTLCWLPFFIVN
hADORA2A   YMVYFNFFACVLVPLLLMLGVYLRIFLAARRQLKQMESQP-------   LPGERARSTLQKEVHAAKSLAIIVGLFALCWLPLHIIN
hCHRM2     AVTFGTAIAAFYLPVIIMTVLYWHISRASKSRIKKDKKEPVANQDPV   KQPAKKKPPPSREKKVTRTILAILLAFIITWAPYNVMV
hHRH1      WFKVMTAIINFYLPTLLMLWFYAKIYKAVRQHCQHRELINRSLPSFS   SRQYVSGLHMNRERKAAKQLGFIMAAFILCWIPYFIFF
hNTSR1     VVIQVNTFMSFIFPMVVISVLNTIIANKLTVMVRQAAEQGQVCTVGG   FSMAIEPGRVQALRHGVRVLRAVVIAFVVCWLPYHVRR
                     TM5                  T4L N-term                T4L C-term              TM6
```

Figure S3 (Shiroishi et al.)

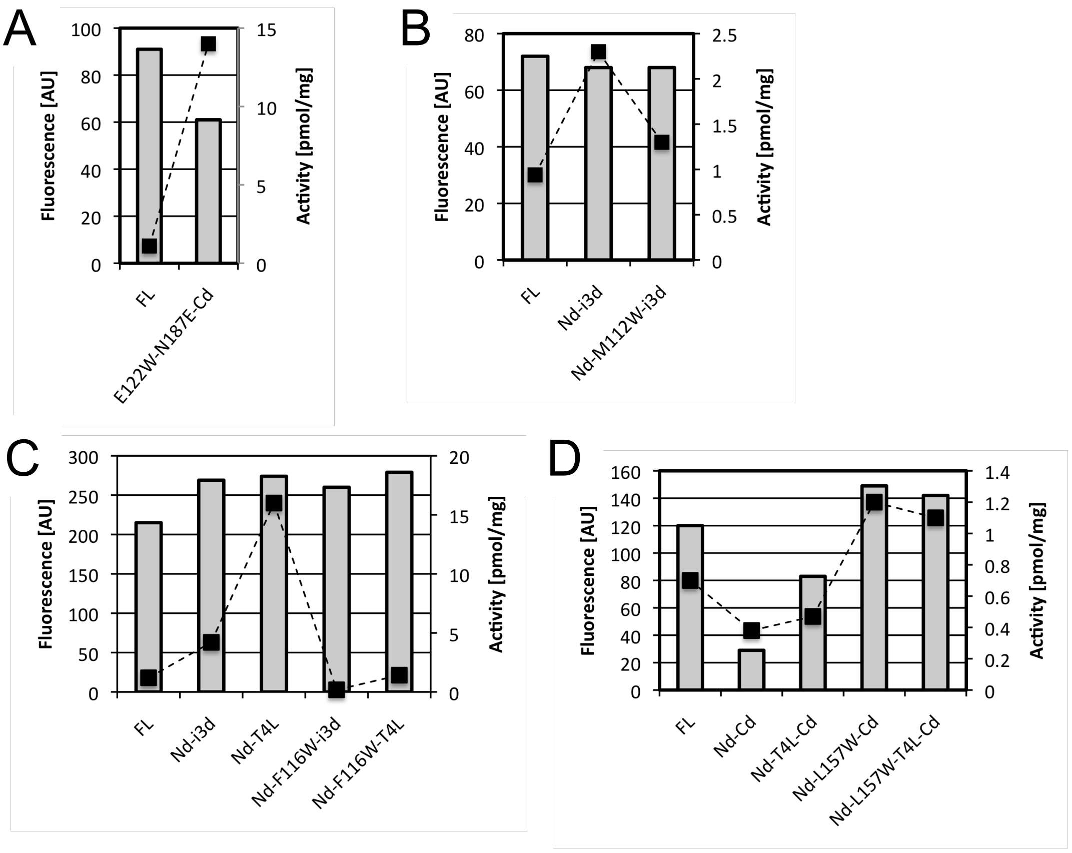

Figure S4 (Shiroishi et al.)

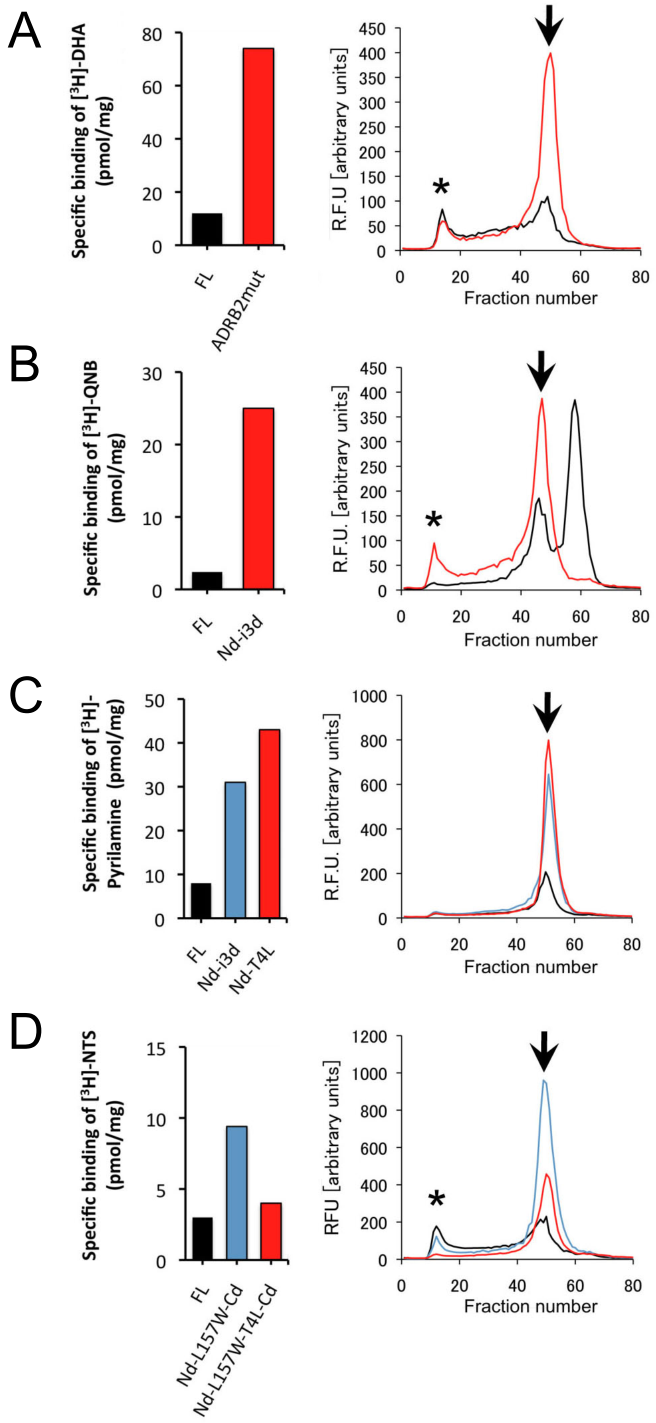

Supplement: Additional file 1 — Supplementary information. Table S1 Primers used for the construction of hHRH1 variant (Nd-F116W-T4L). Table S2 Ligands and conditions for the single point radioligand binding assays. Figure S1 In-gel fluorescence of 25 GPCR-GFP fusions expressed in S. cerevisiae. Arrowheads represent the GPCR-GFP fusion bands and the asterisk represents an endogenous fluorescent ‘background’ protein from S. cerevisiae that migrates at approximately 70 kDa. Figure S2 Construction design of GPCR variants. (A) Sequence alignments of transmembrane 3 (TM3) of the GPCRs in this study with bovine rhodopsin and human ADORA2A. The number above the sequence is the general indexed position based on the Ballesteros–Weinstein system. The 3.41 position for receptor stabilization is highlighted in yellow. (B) Sequence alignments of TM5, i3-loop, and TM6. The position where the T4 lysozyme sequence is fused is shown in red. To truncate the long i3 loop, the residues shown in blue were connected for each receptor. Figure S3 Fluorescence intensity and activity of GPCR variants screened in S. cerevisiae. Whole-cell GFP fluorescence (arbitrary unit, bar graph) and specific activity of the membrane by radioligand binding assays (black square plot) of full-length GPCRs and GPCR variants constructed in S. cerevisiae. (A) hADRB2, (B) hCHRM2, (C) hHRH1, and (D) hNTSR1. Figure S4 Evaluation of the GPCR variants expressed in Sf9 insect cells. The specific binding activities (left) and FSEC profiles (right) of full-length GPCRs and the improved GPCR variants expressed in Sf9 cells are shown. The colors of the chromatogram correspond to those in the binding assays. (A) hADRB2, (B) hCHRM2, (C) hHRH1, (D) hNTSR1. FSEC was performed with a Superose 6 10/300 column. The void peak is denoted by an asterisk. The arrow indicates the target peak of GPCR fused to GFP. [file 1475-2859-11-78-S1.pdf]
